# Clinical and epidemiologic characteristics of inconclusive results in SARS-CoV-2 RT-PCR assays

**DOI:** 10.1186/s12879-021-06534-5

**Published:** 2021-08-21

**Authors:** Yong Kwan Lim, Oh Joo Kweon, Hye Ryoun Kim, Tae-Hyoung Kim, Mi-Kyung Lee

**Affiliations:** 1 Department of Laboratory Medicine, Chung-Ang University College of Medicine, 102 Heukseok-ro, Dongjak-gu, Seoul, 06973 Republic of Korea; 2 Department of Urology, Chung-Ang University College of Medicine, Seoul, Republic of Korea

**Keywords:** SARS-CoV-2, COVID-19, RT-PCR, Inconclusive result, Subgenomic RNA

## Abstract

**Background:**

Inconclusive results in SARS-CoV-2 molecular assays cause confusion among clinicians and delay appropriate infection prevention and control. In this study, we aimed to characterize the respiratory specimens associated with inconclusive SARS-CoV-2 molecular assay results.

**Methods:**

We re-evaluated inconclusive specimens by 3 additional RT-PCR assays and attempted to detect subgenomic RNA (sgRNA) in these specimens.

**Results:**

Among follow-up tests from confirmed SARS-CoV-2 cases, 36.3% of the inconclusive results were classified as presumptive positive results (45/124). However, none of the specimens from 36 screening cases was classified as a presumptive positive result. Among 160 inconclusive specimens, sgRNAs were detected in 78 samples (48.8%): 58 were confirmed cases (58/124, 46.8%) and 20 were screening cases (20/36, 55.6%).

**Conclusions:**

The results of our study suggest the recommendation of considering inconclusive results as positive results for confirmed SARS-CoV-2 cases. In screening cases, viral remnants could be partially amplified in PCR assays, and these inconclusive results could be related to previous infections. In addition, sgRNAs were detected in about half of the inconclusive specimens; however, the clinical significance of sgRNA is not yet clear.

**Supplementary Information:**

The online version contains supplementary material available at 10.1186/s12879-021-06534-5.

## Introduction

On 31 December 2019, the World Health Organization was officially informed of a pneumonia of unknown cause in Wuhan, People's Republic of China, and a new type of coronavirus was isolated on 7 January 2020 [1]. Since then, this novel strain, severe acute respiratory syndrome coronavirus 2 (SARS-CoV-2), has spread rapidly worldwide and caused coronavirus disease 2019 (COVID-19), the respiratory illness responsible for the COVID-19 pandemic [2, 3]. By the end of June 2021, a total of 181 million confirmed cases had occurred worldwide, and more than 3.9 million patients had died from COVID-19 [4].

The molecular assay for detection of SARS-CoV-2 in respiratory specimens is the most essential tool in screening and diagnosing COVID-19, and many countries have allowed the use of in vitro diagnostic devices for this purpose [5–7]. Globally, various real-time reverse transcription–polymerase chain reaction (RT-PCR) assays have been approved for emergency use authorization (EUA) and have shown acceptable performance in many field evaluations [8, 9]. Some assays examine only one gene for detection of SARS-CoV-2; however, others use 2 or more genes [10]. According to protocols, clinical laboratories interpret and report the results of molecular assays.

The Republic of Korea has allowed EUA use of 7 assays based on multiplex real-time RT-PCR for screening and confirming COVID-19 [6], and at least 2 or more of the 4 genes (*E*, *RdRp*, *N*, and *ORF1A*) are used depending on the assay. The Korean Society for Laboratory Medicine (KSLM) and Korea Centers for Disease Control and Prevention (KCDC) had provided guidelines for laboratory diagnosis of COVID-19 [11] and recommended a positive result determination only when all the genes were amplified, even for assays using different genes. Based on this guideline, all kits report positive results only when all the target genes are amplified; however, they report inconclusive results even if 1 target gene is not amplified. Non-amplification of some genes is likely in case of low viral loads in samples and/or could be attributed to different amplification efficiencies of individual target genes during PCR [12–14]. In addition, we suspected that remnant subgenomic RNA (sgRNA) would be amplified in inconclusive specimens. Such inconclusive results always require a re-test, resulting in delays in reporting results and increased testing burden in clinical laboratories. Therefore, the aim of this study was to investigate the characteristics of cases with inconclusive results in COVID-19 molecular assays and re-evaluate inconclusive specimens with another 3 RT-PCR assays approved for EUA. Additionally, we attempted to detect sgRNAs in these specimens.

## Materials and methods

### Patients

This retrospective study included a total of 16,316 samples tested using the SARS-CoV-2 RT-PCR assay from February 19, 2020, to September 30, 2020, at the Chung-Ang University Hospital. The nasopharyngeal/oropharyngeal (NP/OP) swab specimens or sputum specimens were collected for screening asymptomatic individuals who came in close contact of patients with confirmed COVID-19 and for confirming cases of suspected COVID-19. Nucleic acid extraction was performed using the NUCLISENS easyMAG instrument (bioMerieux, Marcy-l'Étoile, France) according to the manufacturer's instructions. The routine SARS-CoV-2 RT-PCR tests were performed with the Allplex™ 2019-nCoV Assay (Seegene Inc., Seoul, Republic of Korea) using the CFX96™ Real-Time PCR Detection System (Bio-Rad, Hercules, CA, USA). The Allplex RT-PCR assay amplifies 3 specific targets: target 1 is the viral *E* gene as a screening test (pan-sarbecovirus target), and targets 2 and 3 are the *RdRP* and *N* genes as a confirmatory test (SARS-CoV-2-specific targets) [11]. In addition, an internal control is used to monitor for the presence of PCR inhibitors in the specimen and therefore to avoid false-negative results. RT-PCR results are reported as “SARS-CoV-2 detected,” “SARS-CoV-2 not detected,” “sarbecovirus detected,” “negative,” “inconclusive,” and “invalid.” If all 3 target genes have been amplified, the result is reported as “SARS-CoV-2 detected.” When only the *E* gene has been amplified, the result is “SARS-CoV-2 not detected, sarbecovirus detected.” Results are considered invalid when the internal control has not been amplified, and “inconclusive” results are reported when only 1 or 2 genes have been amplified. Subsequently, the extracted nucleic acids were stored at –70 ˚C until further analysis. In case of inconclusive Allplex assay results, we re-tested with an increased sample concentration of the stored nucleic acids as recommended in the package insert, and reported the final result.

Among the 16,316 results, a total of 165 specimens from 81 patients were initially reported as inconclusive results, and these specimens were enrolled in our study. For these patients, the following data were collected: basic patient information (age, sex, hospitalization period, mechanical ventilation, and intensive care unit [ICU] admission), symptoms (fever and respiratory symptoms, including cough, sputum, rhinorrhea, and sore throat), days after symptom onset (DASO), and serial Allplex RT-PCR results, when available. This study was approved by the Institutional Review Board of the Chung-Ang University Hospital (IRB no. 2002-017-435). The protocol of this study was performed in accordance with the relevant guidelines and regulations, and the need for informed consent was waived according to the IRB (Institutional Review Board of the Chung-Ang University Hospital) policy.

### RT-PCR for detection of SARS-CoV-2

All samples with inconclusive results after initial routine PCR assays were tested with 3 additional RT-PCR kits authorized for emergency use for diagnosis of COVID-19 in the Republic of Korea: the Real-Q 2019-nCoV Detection Kit (BioSewoom Inc., Seoul, Republic of Korea), BioCore 2019-nCoV Real Time PCR Kit (BioCore Co., Ltd., Seoul, Republic of Korea) and DiaPlexQ Novel Coronavirus (2019-nCoV) Detection Kit (SolGent Co., Ltd., Daejeon, Republic of Korea). Each PCR kit was developed to detect 2 different genes, and the target genes are summarized in Additional file 1: Table S1. These RT-PCR tests were performed using the CFX96™ Real-Time PCR Detection System (Bio-Rad) according to the manufacturer’s specifications. Currently, the KCDC recommends a positive result determination only when all the genes are detected, and all results using these kits included in our study were interpreted according to this recommendation. If only 1 target gene was amplified in 3 comparative RT-PCR assays, we interpreted the result as inconclusive. Each kit included an internal control for assay performance, and invalid results were reported when the internal control was not amplified.

### Subgenomic RNA detection in inconclusive samples

In the nucleic acids extracted for routine RT-PCR testing, we performed additional experiments to detect subgenomic RNA. RNA was reverse transcribed using SuperScript II (Thermo Fisher Scientific, Waltham, MA, USA) and a SARS-CoV-2 specific primer (WHSA-29950R: 5′-TCTCCTAAGAAGCTATTAAAAT-3′) [15, 16]. Then, conventional PCR was carried out with 2 SARS-CoV-2-specific primers (WHSA-00025F: 5′-CCAACCAACTTTCGATCTCTTGTA-3′ and WHSA-29925R: 5′-ATGGGGATAGCACTACTAAAATTA-3′). These primers targeted the common 5′ leader sequence and the 3' untranslated region; although the sizes of sgRNA amplicons were expected to vary depending on the 5' and 3' breakpoints, they were always shorter than the size of SARS-CoV-2 genome amplicons (< 30,000 bp) [17, 18]. Thermal cycling included 95 °C for 3 min followed by 40 cycles of 94 °C for 30 s, 56 °C for 30 s, and 72 °C for 1.5 min. The PCR products were subjected to 1% agarose gel electrophoresis.

### Statistics

Because there is no reference method for SARS-CoV-2 detection, we assumed that existence of viral RNA was inferred through the results of PCR assays. Samples with positive results in more than 50% of RT-PCR assays were considered presumptive positive specimens, and other specimens were considered presumptive negative. For assessing the reliability of agreement among the 4 SARS-CoV-2 RT-PCR assays, Fleiss' kappa coefficients were calculated and assessed according to the following criteria: 0.81–1.00 for almost perfect agreement, 0.61–0.80 for substantial agreement, 0.41–0.60 for moderate agreement, 0.21–0.40 for fair agreement, 0.00–0.20 for slight agreement, and < 0.00 for poor agreement [19]. Categorical and continuous variables were compared using the Chi-square test and Wilcoxon signed rank test, respectively. A *p*-value < 0.05 was considered a significant difference. With serial RT-PCR results in confirmed cases, we performed probit regression models to investigate changes in dichotomous RT-PCR and target gene results according to DASO. Using probit analysis, we could estimate when more than 95% of the RT-PCR and target gene results were negative. All statistical analyses were performed with R version 4.0.3 (http://www.R-project.org/).

## Results

A total of 16,316 tests were performed between Feb 2020 and Sep 2020, and 165 results were initially reported as inconclusive. One hundred and twenty-five inconclusive results came from 41 confirmed SARS-CoV-2 cases that were first diagnosed or underwent follow-up tests, and 40 results were derived from screening asymptomatic individuals (Table 1). None of the enrolled patients had to be admitted to the ICU or required mechanical ventilation. In addition, no patients died during treatment. The initial amplification characteristics of the inconclusive results are summarized in Table 2. Only one gene (*RdRp* or *N*) was detected in 61.6% and 87.5% of confirmed cases and screening cases, respectively, and the rest of the specimens were positive for 2 genes. Interestingly, the *N* gene was initially detected in almost all specimens with inconclusive results (98.4% and 92.5% for confirmed and screening cases, respectively).

**Table 1:** Characteristics of patients initially reported as having inconclusive results

	Confirmed cases $(n=41)$	Screening cases $(n=40)$
Age (Q1–Q3)^a^	62.0 (40.5–69.0)	50.0 (26.3–64.5)
Male	26 (63.4%)	20 (50.0%)
Symptom		
Fever	31 (75.6%)	0 (0%)
Respiratory	22 (53.7%)	0 (0%)
Asymptomatic	5 (12.2%)	40 (100%)
Days after symptom onset (Q1–Q3)^a^	15 (11–21)	N/A
Length of hospital stay (Q1–Q3)^a^	16 (14–21)	N/A

^a^The results are presented as medians with interquartile ranges

N/A, not applicable

**Table 2:** Amplification result for each gene for inconclusive results using the Allplex™ 2019-nCoV assay (initial result)

Targeted gene	Specimens from confirmed cases (*n* = 125)	Specimens from screening cases (*n* = 40)
*RdRp* gene only (%)	2 (1.6%)	3 (7.5%)
*N* gene only (%)	75 (60.0%)	32 (80.0%)
*E* gene and *N* gene (%)	5 (4.0%)	1 (2.5%)
*RdRp* gene and *N* gene (%)	43 (34.4%)	4 (10.0%)

To determine the actual results of initially inconclusive results, we re-tested the samples using the Allplex assay and performed an additional 3 RT-PCR tests on the same RNA samples. Among 165 specimens, 5 (1 confirmed case and 4 screening cases) were excluded from additional tests because of insufficient sample quantities. According to the 4 RT-PCR results, we classified the inconclusive specimens as presumptive positive/negative results. The results of the 4 RT-PCR assays are summarized in Table 3. Among 124 specimens from confirmed cases, 45 specimens were classified as presumptive positive specimens (36.3%). However, none of the specimens from the 36 screening cases was classified as a presumptive positive result. When considering the degree of agreement between the 4 assays (Fleiss' kappa), there was very low concordance (poor agreement) between the results in both the presumptive positive and presumptive negative specimens from confirmed and screening cases. The amplification results and cycle threshold (Ct) values of each gene are summarized in Table 4. The average Ct value for each target gene ranged from 34.6‒36.4 for the *E* gene, 32.0‒38.5 for the *RdRP* gene, and 37.1‒38.4 for the *N* gene; it was 37.3 for the *ORF1a* gene. These results showed very high Ct values, close to the cutoff Ct values.

**Table 3:** SARS-CoV-2 RT-PCR results in presumptive positive and negative specimens

Presumptive results	RT-PCR results	Allplex (re-test)	Real-Q	BioCore	DiaPlexQ	Fleiss’ κ
Specimens from confirmed cases $(n=124)$						
Presumptive positive (45/124, 36.3%)	Positive	16 (35.6%)	40 (88.9%)	9 (20%)	43 (95.6%)	− 0.176
	Inconclusive	28 (62.2%)	5 (11.1%)	29 (64.4%)	2 (4.4%)	
	Negative	1 (2.2%)	0 (0%)	7 (15.6%)	0 (0%)	
Presumptive negative (79/124, 63.7%)	Positive	2 (2.5%)	4 (5.1%)	1 (1.3%)	28 (35.4%)	− 0.001
	Inconclusive	49 (62.0%)	21 (26.6%)	59 (74.7%)	23 (29.2%)	
	Negative	28 (35.5%)	54 (68.3%)	19 (24.0%)	28 (35.4%)	
Specimens from screening cases $(n=36)$						
Presumptive negative (36/36, 100%)	Positive	0 (0%)	0 (0%)	0 (0%)	0 (0%)	− 0.001
	Inconclusive	15 (41.7%)	3 (8.3%)	2 (5.6%)	0 (0%)	
	Negative	21 (58.3%)	33 (91.7%)	34 (94.4%)	36 (100%)

**Table 4:** Amplification results for each gene in specimens from confirmed cases and screening cases

	Specimens from confirmed cases		Specimens from screening cases	
	Detection rate (%)	Cycle threshold (SD)	Detection rate (%)	Cycle threshold (SD)
*E* gene				
Allplex (initial)	5/125 (4.0%)	36.0 (2.1)	1/40 (2.5%)	35.7 ( −)
Allplex (re-test)	19/124 (15.3%)	34.6 (1.5)	0/36 (0%)	N/A
Real-Q	60/124 (48.4%)	36.4 (1.1)	0/36 (0%)	N/A
*RdRP* gene				
Allplex (initial)	45/125 (36.0%)	35.8 (1.7)	7/40 (17.5%)	38.0 (1.6)
Allplex (re-test)	52/124 (41.9%)	35.6 (1.6)	4/36 (11.1%)	36.6 (2.4)
Real-Q	54/124 (43.5%)	36.2 (1.3)	3/36 (8.3%)	32.0 (4.8)
BioCore	55/124 (44.4%)	37.1 (1.7)	2/36 (5.6%)	38.5 (0.8)
*N* gene				
Allplex (initial)	123/125 (98.4%)	37.3 (1.6)	37/40 (92.5%)	38.4 (1.2)
Allplex (re-test)	92/124 (74.2%)	37.1 (1.6)	15/36 (41.7%)	37.5 (1.3)
BioCore	11/124 (8.9%)	38.3 (0.7)	0/36 (0%)	N/A
DiaPlexQ	85/124 (68.5%)	37.3 (1.3)	0/36 (0%)	N/A
*ORF1a*				
DiaPlexQ	81/124 (65.3%)	37.3 (1.2)	0/36 (0%)	N/A

N/A, not applicable

**Fig. 1:**
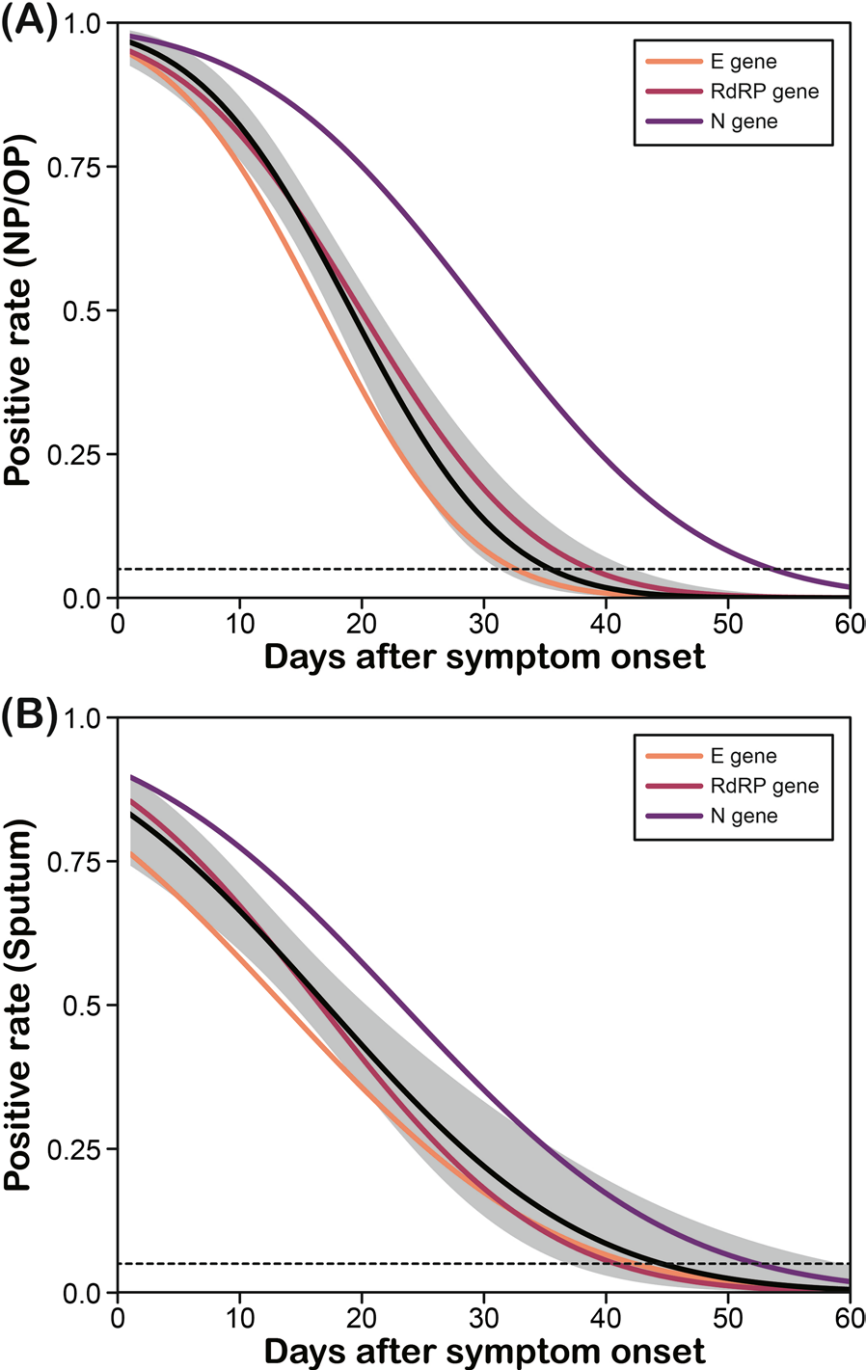
Probability curves of PCR results versus days after symptom onset for NP/OP swab specimens (**A**) and sputum samples (**B**) from confirmed COVID-19 cases. The black solid lines and shaded areas depict the estimated probabilities of positive RT-PCR results and their 95% confidence intervals; the colored solid lines depict the estimated positive rates for the 3 target genes (*E*, *RdRP*, and *N*) included in the Allplex kit. The horizontal dotted line indicates that more than 95% of the specimens were negative

Forty-one confirmed patients underwent a total of 564 tests during hospitalization. Among these, 299 were NP/OP swab specimens, and 264 were sputum specimens. The RT-PCR test was performed at a median of 16 days (Q1–Q3: 11–21) after symptom onset, and the maximum DASO was 60. In probit analyses, the time points when 95% of the RT-PCR results became negative were 35.4 (NP/OP swab specimens) and 40.4 (sputum specimens) days (Fig. 1). The time points for each gene were 32.6 (*E*), 38.8 (*RdRP*), and 53.6 (*N*) days and 42.4 (*E*), 44.5 (*RdRP*), and 52.4 (*N*) days for NP/OP and sputum specimens, respectively.

The results of sgRNA detection on the inconclusive specimens are summarized in Table 5. Among 160 inconclusive specimens, sgRNAs were detected in 78 samples (48.8%): 58 samples were from confirmed cases (58/124, 46.8%), and 20 were from screening cases (20/36, 55.6%). We divided the sgRNA-positive and sgRNA-negative groups from the specimens of confirmed cases and compared symptoms, DASO, and the 4 RT-PCR results; however, there were no significantly different results between the two groups.

**Table 5:** Results of subgenomic RNA (sgRNA) detection on inconclusive specimens from confirmed cases and screening cases

	Specimens from confirmed cases (*n* = 124)			Specimens from screening cases (*n* = 36)		
	sgRNA positive	sgRNA negative	*p*-value	sgRNA positive	sgRNA negative	*p*-value
Patients (*n*)	58	66		20	16	
Symptom						
Fever	14 (24.1%)	17 (25.8%)	1	0 (0%)	0 (0%)	1
Respiratory symptoms	9 (15.6%)	14 (21.2%)	0.491	0 (0%)	0 (0%)	1
Asymptomatic	38 (65.6%)	43 (65.2%)	1	20 (100%)	16(100%)	1
Days after symptom onset						
Median (Q1–Q3)^a^	16 (10 – 19)	17 (13 – 22)	0.216	N/A	N/A	
Min	1	3		N/A	N/A	
Max	43	49		N/A	N/A	
Specimens						
NP/OP	34 (58.6%)	37 (56.1%)	0.774	20 (100%)	16 (100%)	1
Sputum	24 (41.4%)	29 (43.9%)				
Presumptive result						
Positive	23 (39.7%)	22 (33.3%)	0.465	0 (0%)	0 (0%)	1
Negative	35 (60.3%)	44 (66.7%)		20 (100%)	16 (100%)	
Each RT-PCR result						
Allplex (re-test)						
Positive	7 (12.1%)	11 (16.7%)	0.623	0 (0%)	0 (0%)	0.741
Inconclusive	38 (65.5%)	38 (57.6%)		9 (45.0%)	6 (37.5%)	
Negative	13 (22.4%)	17 (25.8%)		11 (55.0%)	10 (62.5%)	
Real-Q						
Positive	26 (44.8%)	18 (27.3%)	0.129	0 (0%)	0 (0%)	1
Inconclusive	11 (19.0%)	15 (22.7%)		2 (10.0%)	1 (6.3%)	
Negative	21 (36.2%)	33 (50.0%)		18 (90.0%)	15 (93.8%)	
BioCore						
Positive	5 (8.6%)	5 (7.6%)	0.704	0 (0%)	0 (0%)	1
Inconclusive	20 (34.5%)	28 (42.4%)		1 (5.0%)	1 (6.3%)	
Negative	33 (56.9%)	33 (50.0%)		19 (95.0%)	15 (93.8%)	
DiaPlexQ						
Positive	32 (55.2%)	39 (59.1%)	0.123	0 (0%)	0 (0%)	1
Inconclusive	16 (27.6%)	9 (13.6%)		0 (0%)	0 (0%)	
Negative	10 (17.2%)	18 (27.3%)		20 (100.0%)	16 (100.0%)	

^a^The results are presented as medians with interquartile ranges

NP/OP, nasopharyngeal/oropharyngeal swab specimens

## Discussion

Early diagnosis of SARS-CoV-2 infection is one of the most important factors for infection prevention and control [20]. Many commercially available RT-PCR assays have been developed to detect multiple target genes, and differences in interpretation of the RT-PCR results exist, depending on the circumstances of each country. For example, a positive result could be inferred on detection of all target genes or when only one target gene is detected [7, 11]. Basis the interpretation criteria, these tests may inevitably yield some inconclusive results, and these results cause confusion among clinicians and delay appropriate infection prevention and control [21].

In our study, most of the inconclusive RT-PCR results were associated with high frequencies of positive results for the *N* gene. Out of the 165 inconclusive results, the *N* gene was detected in 160 of them. For screening cases, the *N* gene was detected in 37 samples out of 40 inconclusive results. Although none of the inconclusive results from the screening cases were designated presumptive positive in additional PCR assays, we suspected that the inconclusive results from screening cases would be related to past asymptomatic infection and the RT-PCR amplification of viral RNA remnants. In the probit analysis of confirmed COVID-19 cases, the *N* gene was detected for about 2 weeks, even if the PCR result changed to negative; this phenomenon would contribute to inconclusive results in which the *N* gene was detected.

When comparing the results of RT–PCR assays in the inconclusive specimens, there were very low concordances in the confirmed and screening cases. SARS-CoV-2 RT-PCR assays are known to be affected by various sources of variability, such as sampling methods of NP/OP swab, RNA extraction methods, and lot-to-lot variation in RT-PCR kits. However, the test results could also be affected by several limitations of multiplex PCR assays, including varying amplification efficiencies of target genes and false-negative signals caused by inhibitors [9, 12]. In addition, in the Republic of Korea, a positive result is only reported when all target genes have been amplified, according to KCDC guidelines [11]. Therefore, in specimens with extremely small viral RNA loads, an inconclusive result is expected, and the phenomenon of low concordance between various PCR assays would persist because of the previously described limitations of multiplex PCR assays and the KCDC guidelines. Therefore, efforts for standardization would be needed for the inclusion of target genes and the amplification efficiencies of each target gene in SARS-CoV-2 multiplex RT-PCR assays. In addition, although most of the inconclusive results had Ct values exceeding 35 for each target gene, it cannot be concluded that a patient with an inconclusive result is not infectious [22–24], and these results should be considered positive, especially in the follow-up results from confirmed COVID-19 cases.

In our study, SARS-CoV-2 sgRNA was found in half of the inconclusive specimens. Coronavirus sgRNAs are thought to encode various proteins, including the spike (S), envelope (E), membrane (M), nucleocapsid (N), and several accessory proteins [18]. It is hypothesized that replication of sgRNAs is associated with the double-membrane vesicles in the cytoplasm of infected cells, which protect the virus from host-cell recognition and responses [17]. For follow-up test results of confirmed COVID-19 cases, even if full-length viral genomic RNA did not remain, we suspected that some remaining sgRNAs had been detected in the specimens by the routine molecular assays, resulting in inconclusive results for up to 43 days after symptom onset. In addition, sgRNAs were detected in more than half of the samples from the screening cases, and these results may imply that the inconclusive results from asymptomatic screening patients indicate previous SARS-CoV-2 infection. However, we could not determine the clinical significance of sgRNA in respiratory specimens, and whether sgRNA detection has diagnostic value requires further study.

Despite significant results demonstrating the characteristics of inconclusive results of the COVID-19 PCR assay, there were several limitations to our study. First, it was not possible to determine if a specimen with an inconclusive result could actually be infectious. In addition, we could not confirm that inconclusive specimens or specimens containing sgRNAs represents a previous infection. Second, we could not carry out serologic assays in parallel with the RT-PCR assays. Availability of antibody test results would have helped determine whether the screening cases indeed had previous infections.

In conclusion, because of technical limitations and the interpretation criteria for molecular assays, inconclusive SARS-CoV-2 RT-PCR assay results are inevitable, and these results would be continual. When an inconclusive result came from a confirmed COVID-19 case, 36.3% of the inconclusive results would be classified as positive results; we would recommend considering these results as positive because of any possibility of infectiousness. In the case of screening cases, an inconclusive result would suggest past infection, and we would recommend a SARS-CoV-2 antibody assay. In addition, sgRNAs were detected in about half of inconclusive specimens; however, the clinical significance of sgRNA is not yet clear, and further long-term study should be performed to gain better insight into the clinical role of sgRNA in SARS-CoV-2 infections.

## Supplementary Information

**Additional file 1: Table S1.** Target genes of 4 real-time RT–PCR assays.

## Data Availability

The datasets generated and analyzed during the current study are not publicly available due to the IRB policy, however are available from the corresponding author on reasonable request.

## References

[1] World Health Organization. Novel Coronavirus (2019-nCoV): situation report, 1. 2020.

[2] Hu B, Guo H, Zhou P, Shi ZL (2021). Characteristics of SARS-CoV-2 and COVID-19. Nat Rev Microbiol.

[3] Kweon OJ, Lim YK, Kim HR, Kim MC, Choi SH, Chung JW, Lee MK (2020). Antibody kinetics and serologic profiles of SARS-CoV-2 infection using two serologic assays. PLoS One.

[4] World Health Organization. WHO Coronavirus Disease (COVID-19) Dashboard. 2021. (https://covid19.who.int/). 37184163

[5] U.S. Food and Drug Administration. In Vitro Diagnostics EUAs. 2021. https://www.fda.gov/medical-devices/coronavirus-disease-2019-covid-19-emergency-use-authorizations-medical-devices/vitro-diagnostics-euas#individual-molecular.

[6] Ministry of Food and Drug Safety. Ministry of Food and Drug Safety approves emergency use of COVID-19 diagnostic reagents. 2021. (https://www.mfds.go.kr/eng/brd/m_64/view.do?seq=34&srchFr=&srchTo=&srchWord=&srchTp=&itm_seq_1=0&itm_seq_2=0&multi_itm_seq=0&company_cd=&company_nm=&page=1).

[7] Freire-Paspuel B, Bruno A, Orlando A, Garcia-Bereguiain MA (2021). Analytical and clinical evaluation of two RT-qPCR SARS-CoV-2 diagnostic tests with emergency use authorization in Ecuador. Am J Trop Med Hygiene.

[8] Green DA, Zucker J, Westblade LF, Whittier S, Rennert H, Velu P, Craney A, Cushing M, Liu D, Sobieszczyk ME, et al. Clinical performance of SARS-CoV-2 molecular tests. J Clin Microbiol. 2020;58(8):e00995–20. 10.1128/JCM.00995-20 PMC7383556 32513858

[9] Hur KH, Park K, Lim Y, Jeong YS, Sung H, Kim MN. Evaluation of four commercial kits for SARS-CoV-2 real-time reversetranscription polymerase chain reaction approved by emergency-use-authorization in Korea. Front Med (Lausanne). 2020;7:521. 10.3389/fmed.2020.00521 PMC7438443 32903503

[10] Kim A, Lee H, Hur KW, Sung H, Kim M-N (2020). Causes and clinical relevance of inconclusive SARS-CoV-2 real-time reverse transcriptionPCR test results. Ann Clin Microbiol.

[11] Hong KH, Lee SW, Kim TS, Huh HJ, Lee J, Kim SY, Park JS, Kim GJ, Sung H, Roh KH (2020). Guidelines for laboratory diagnosis of coronavirus disease 2019 (COVID-19) in Korea. Ann Lab Med.

[12] Bhattacharya S, Vidyadharan A, Joy VM (2020). Inconclusive SARS-COV-2 reverse transcription-polymerase chain reaction test reports: interpretation, clinical and infection control implications. J Acad Clin Microbiol.

[13] Freire-Paspuel B, Vega-Marino P, Velez A, Cruz M, Perez F, Garcia-Bereguiain MA (2021). Analytical and clinical comparison of Viasure (CerTest Biotec) and 2019-nCoV CDC (IDT) RT-qPCR kits for SARS-CoV2 diagnosis. Virology.

[14] Freire-Paspuel B, Vega-Marino P, Velez A, Castillo P, Cruz M, Garcia-Bereguiain MA (2020). Evaluation of nCoV-QS (MiCo BioMed) for RT-qPCR detection of SARS-CoV-2 from nasopharyngeal samples using CDC FDA EUA qPCR kit as a gold standard: an example of the need of validation studies. J Clin Virol.

[15] Perera R, Tso E, Tsang OTY, Tsang DNC, Fung K, Leung YWY, Chin AWH, Chu DKW, Cheng SMS, Poon LLM (2020). SARS-CoV-2 virus culture and subgenomic RNA for respiratory specimens from patients with mild coronavirus disease. Emerg Infect Dis.

[16] Simons FA, Vennema H, Rofina JE, Pol JM, Horzinek MC, Rottier PJ, Egberink HF (2005). A mRNA PCR for the diagnosis of feline infectious peritonitis. J Virol Methods.

[17] Alexandersen S, Chamings A, Bhatta TR (2020). SARS-CoV-2 genomic and subgenomic RNAs in diagnostic samples are not an indicator of active replication. Nat Commun.

[18] Kim D, Lee JY, Yang JS, Kim JW, Kim VN, Chang H (2020). The architecture of SARS-CoV-2 transcriptome. Cell.

[19] Landis JR, Koch GG (1977). The measurement of observer agreement for categorical data. Biometrics.

[20] Chen N, Zhou M, Dong X, Qu J, Gong F, Han Y, Qiu Y, Wang J, Liu Y, Wei Y (2020). Epidemiological and clinical characteristics of 99 cases of 2019 novel coronavirus pneumonia in Wuhan, China: a descriptive study. Lancet.

[21] Yang S, Stanzione N, Uslan DZ, Garner OB, de St Maurice A (2020). Clinical and epidemiologic evaluation of inconclusive COVID-19 PCR results using a quantitative algorithm. Am J Clin Pathol.

[22] Avanzato VA, Matson MJ, Seifert SN, Pryce R, Williamson BN, Anzick SL, Barbian K, Judson SD, Fischer ER, Martens C (2020). Case study: prolonged infectious SARS-CoV-2 shedding from an asymptomatic immunocompromised individual with cancer. Cell.

[23] Walsh KA, Jordan K, Clyne B, Rohde D, Drummond L, Byrne P, Ahern S, Carty PG, O'Brien KK, O'Murchu E (2020). SARS-CoV-2 detection, viral load and infectivity over the course of an infection. J Infect.

[24] Rhee C, Kanjilal S, Baker M, Klompas M. Duration of severe acute respiratory syndrome coronavirus 2 (SARS-CoV-2) infectivity: when is it safe to discontinue isolation? Clin Infect Dis. 2020;72(8):1467–74. 10.1093/cid/ciaa1249 PMC7499497 33029620

