# Supplementary material for: Clinical and epidemiologic characteristics of inconclusive results in SARS-CoV-2 RT-PCR assays
Source: BMC Infect Dis. 2021 Aug 21;21:851. doi: 10.1186/s12879-021-06534-5 (PMC8379569; doi:10.1186/s12879-021-06534-5)
Supplement: Supplementary file 1 — Additional file 1: Table S1. Target genes of 4 real-time RT–PCR assays. [file 12879_2021_6534_MOESM1_ESM.docx]

**Table S1.** Target genes of 4 real-time RT–PCR assays

| Kit name | Manufacturer | Target genes | Cut-off of Ct value | Claimed limit of detection |
| --- | --- | --- | --- | --- |
| Allplex™ 2019-nCoV Assay | Seegene Inc., Republic of Korea | *E, RdRp, N* | *E, RdRp, N* and IC ≤ 40 | 100 RNA copies/reaction |
| Real-Q 2019-nCoV Detection Kit | BioSewoom Inc., Republic of Korea | *E, RdRp* | *E* and *RdRp* ≤ 38, IC ≤ 40 | 38-40 copies of *E* gene/reaction  33-34 copies of *RdRp* gene/reaction |
| BioCore 2019-nCoV Real Time PCR Kit | BioCore Co. Ltd., Republic of Korea | *RdRp, N* | *RdRp, N* and IC ≤ 40 | 630 RNA copies/reaction |
| DiaPlexQ Novel Coronavirus (2019-nCoV) Detection Kit | SolGent Co. Ltd., Republic of Korea | *N, ORF1a* | *N* and *ORF1a* ≤ 40, IC ≤ 26 | 10 RNA copies/reaction |

Ct, cycle threshold; IC, internal control
